# Early initial video-electro-encephalography combined with variant location predict prognosis of KCNQ2-related disorder

**DOI:** 10.1186/s12887-021-02946-z

**Published:** 2021-10-28

**Authors:** Yan Xu, Ya-lan Dou, Xiang Chen, Xin-ran Dong, Xin-hua Wang, Bing-bing Wu, Guo-qiang Cheng, Yuan-feng Zhou

**Affiliations:** 1 Department of Neurology, Children’s Hospital of Fudan University, National Children’s Medical Center, NO.399 Wanyuan Road, Minhang District, Shanghai, 201102 China; 2 Department of clinical Epidemiology, Children’s Hospital of Fudan University, National Children’s Medical Center, Shanghai, China; 3 Department of Neonatology, Children’s Hospital of Fudan University, National Children’s Medical Center, NO.399 Wanyuan Road, Minhang District, Shanghai, 201102 China; 4 Molecular Medical Center, Children’s Hospital of Fudan University, National Children’s Medical Center, Shanghai, China; 5 The Molecular Genetic Diagnosis Center, Shanghai Key Lab of Birth Defects, Pediatrics Research Institute, Children’s Hospital of Fudan University, National Children’s Medical Center, Shanghai, China

**Keywords:** *KCNQ2*-related disorder, Neonate, Multivariate ordinal logistic regression analysis, Prognosis, Video-electro-encephalography, Variant location

## Abstract

**Background:**

The clinical features of KCNQ2-related disorders range from benign familial neonatal seizures 1 to early infantile epileptic encephalopathy 7. The genotype-phenotypic association is difficult to establish.

**Objective:**

To explore potential factors in neonatal period that can predict the prognosis of neonates with *KCNQ2*-related disorder.

**Methods:**

Infants with *KCNQ2*-related disorder were retrospectively enrolled in our study in Children’s Hospital of Fudan University in China from Jan 2015 to Mar 2020. All infants were older than age of 12 months at time of follow-up, and assessed by Bayley Scales of Infant and Toddler Development-Third Edition (BSID-III) or Wechsler preschool and primary scale of intelligence-fourth edition (WPPSI-IV), then divided into three groups based on scores of BSID-III or WPPSI-IV: normal group, mild impairment group, encephalopathy group. We collected demographic variables, clinical characteristics, neuroimaging data. Considered variables include gender, gestational age, birth weight, age of the initial seizures, early interictal VEEG, variant location, delivery type. Variables predicting prognosis were identified using multivariate ordinal logistic regression analysis.

**Results:**

A total of 52 infants were selected in this study. Early interictal video-electro-encephalography (VEEG) (β = 2.77, 1.20 to 4.34, *P* = 0.001), and variant location (β = 2.77, 0.03 to 5.5, *P* = 0.048) were independent risk factors for prognosis. The worse the early interictal VEEG, the worse the prognosis. Patients with variants located in the pore-lining domain or S4 segment are more likely to have a poor prognosis.

**Conclusions:**

The integration of early initial VEEG and variant location can predict prognosis. An individual whose *KCNQ2* variant located in voltage sensor, the pore domain, with worse early initial VEEG background, often had an adverse outcome.

## Background

*KCNQ2* encodes a voltage-gated potassium channel protein, reciprocally with *KCNQ3* to form a potassium channel with essentially identical properties to the channel underlying the native M-current. *KCNQ2***-**related disorders represent a broad continuum of epileptic phenotypes caused by a heterozygous variant in *KCNQ2*. The clinical features of *KCNQ2*-related disorders range from mild forms, as benign familial neonatal seizures 1 (BFNS1, [MIM:121200]), to very severe ones, as early infantile epileptic encephalopathy 7 (EIEE7, [MIM:613720]). *KCNQ2*-related disorder is generally characterized by multiple daily seizures that usually occurs between the first to eighth day of life [1–12], rare cases at few months of life [4–7]. Seizure types are mostly tonic or apneic episodes, focal clonic activity, or autonomic changes [1–12].

Generally, individuals with identical pathogenic variants appear similar clinical features and developmental outcomes, but some cases exhibit clinical heterogeneity. For example, one or more individuals who developed a therapy-resistant epileptic encephalopathy have been observed in families with BFNS1 [13–15]; the p.Arg213Trp *KCNQ2* pathogenic variant has been reported in an individual with severe epileptic encephalopathy [16] and in another family with normal outcome [17]. Heterogeneity of phenotype indicated the complex correlation with genotype.

Limited by number of cases, few studies focus on the relationship between clinical features, treatment and prognosis of *KCNQ2*-related disorder [18, 19]. We are seeking for some factors in neonatal period that can predict the long-term outcome, such as: interictal video-electro-encephalography (VEEG), date of onset, variant location, and so on.

## Methods

### Patients

We retrospectively recruited the infants who had seizures within the first month of life in Children’s Hospital of Fudan University in China from Jan 2015 to Mar 2020. Of all the infants with convulsions, we selected those have *KCNQ2* gene variants identified by molecular genetic testing and agreed to have VEEG examination during the neonatal period in our study. They were followed up to at least 12 months of age.

### Genetic testing

Genomic DNA were extracted from peripheral blood. Whole exome sequencing (WES) or clinical exome sequencing (2742 targed genes) was performed to identify mutations. We used public databases (the dbSNP137 reported in the UCSC Genome Browser, the exome aggregation consortium, and the 1000 Genome Project) and our local database to filter the variants. Sanger sequencing was performed to confirm the candidate variants. In this cohort, patients enrolled were met one of the following five criteria: 1) patients with a previously established heterozygous pathogenic variant in KCNQ2 gene, which from both public database and internal database; 2) same amino acid change as a previously established pathogenic variant, however different nucleotide change; 3) patients with novel (both public database and internal database) heterozygous null variant (nonsense, frameshift, canonical +/− 1 or 2 splice sites, initiation codon) in KCNQ2 gene; 4) patients with heterozygous variant, which is neither in public database nor in internal database, but is a de novo variant; 5) patients with heterozygous variant inherited from the affected parent. All the genetic results were interpreted by two experienced molecular genetic clinicians simultaneously.

It is well known that KCNQ2 gene share a common design of four α subunits, each α subunit contains six transmembrane segments, with cytoplasmic N-terminal (short) and C-terminal (long) regions, S1–S4 forming the voltage sensor and S5–S6 forming the pore-lining domain [20, 21]. S4 plays as the main sensor for depolarization [20–22]. Variants associated with epileptic encephalopathy and located in the pore region or S4 segment produce dominant-negative effects and reduce current density by 50–70% [23, 24].

We selected S4 segment and the pore-lining domain (from S5 to S6, including S5,S5-H5 linker, H5,H5-S6 linker, S6) as key spots.

We categorized the variant location of *KCNQ2*: 0, non-key spots; 1, key spots.

### VEEG

Scalp EEGs were recorded at 8 electrodes (F3/F4, C3/C4, T3/T4, P3/P4,) according to the international 10–20 system using the Nicolet VEEG monitor (Nicoletone, Middleton, Wisconsin, United States). Each neonate had at least one VEEG recording. Each recording lasted more than 4 h including at least one full cycle of wakefulness and sleep.

Most infants have been treated with anti-epileptic drugs (AEDs) in other hospitals. In order to reduce the deviation caused by AEDs, for the infants who have not used anticonvulsant drugs before, we use the VEEG data of more than 3 days after taking AEDs in our hospital as early interictal VEEG, while used the first VEEG data in our hospital for the patient who have been treated with AEDs in other hospital as early interictal VEEG.

Each VEEG data was read by two experienced neurophysiologists individually. If the results from the two neurophysiologists were consistent, they would directly apply it. If not, they would discuss together to give the final results. If they can’t reach an agreement in the end, the data would be discussed by the VEEG team which including more than three more experienced neurophysiologists.

According to interictal VEEG classification, we divide VEEG into normal, mild abnormal, and severe abnormal [25]. VEEG that does not meet the above three conditions was divided into two groups according to whether the sleep cycle can be distinguished.

**Fig. 1:**
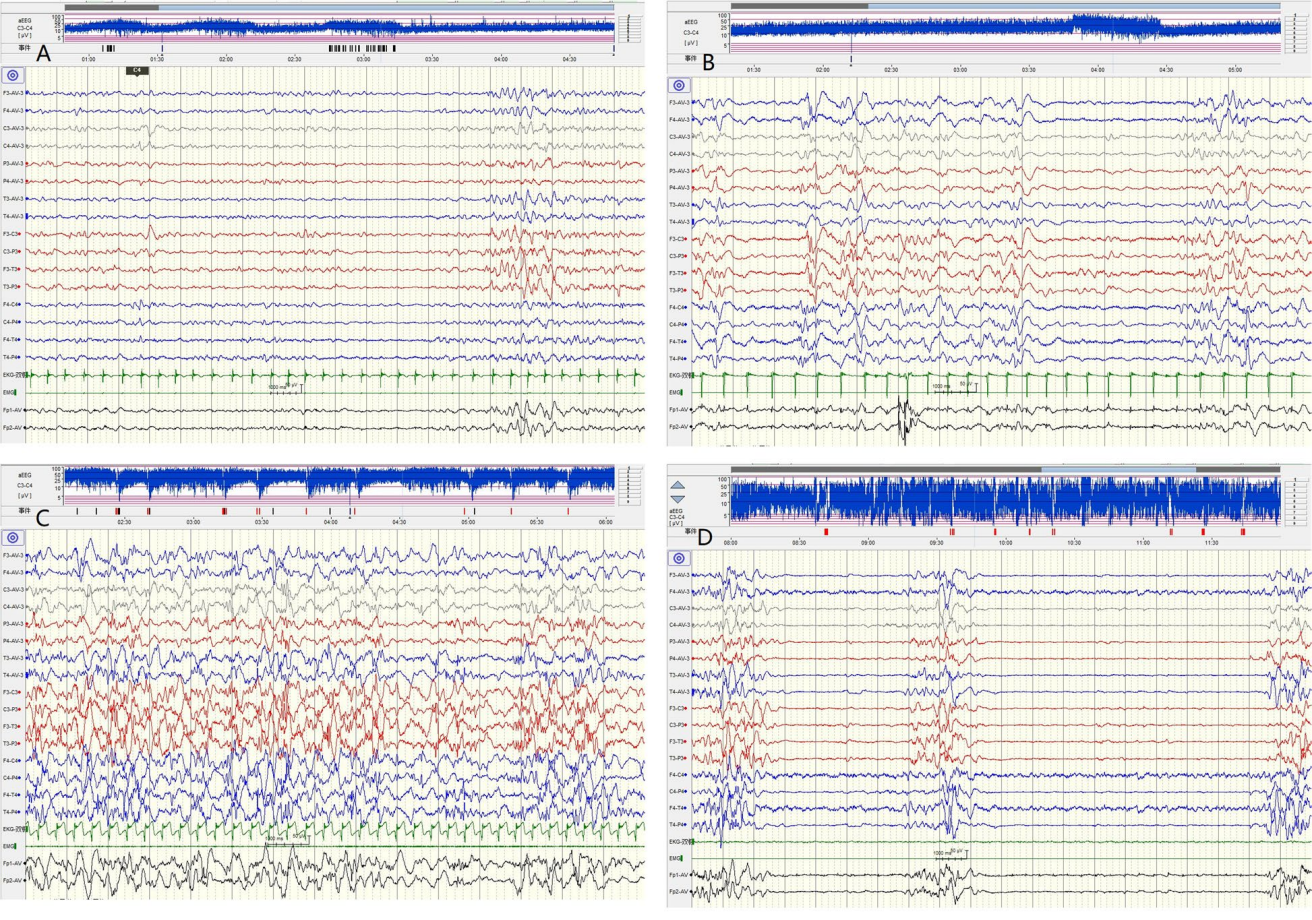
**A**: Patient 8, mildly abnormal: mild multifocal sharp waves. **B**: Patient 5, moderately abnormal: multifocal sharp waves, with sleep cycle. **C**: Patient 2, moderately abnormal: multifocal sharp waves, without sleep cycle. **D**: Patient 29, markedly abnormal: burst-suppression pattern

We assigned points according to the following conditions:

0, normal
1, Mildly abnormal: mild multifocal sharp waves (Fig. 1.A);
2, Moderately abnormal: multifocal sharp waves, with sleep cycle (Fig. 1.B);
3, Moderately abnormal: multifocal sharp waves, without sleep cycle (Fig. 1.C);
4, Markedly abnormal: burst-suppression pattern (Fig. 1.D).

### Delivery type

0, Vaginal delivery; 1, Cesarean section.

### Other clinical data

Clinical manifestations, including gender, birth weight, age of the onset, and gestational age, of each proband were ascertained comprehensively through physician by the review of the medical records.

### Outcome

Prognosis referred to the prognosis of at least 12 months of follow-up. All infants were older than 12 months old at time of follow-up. The Bayley Scales of Infant and Toddler Development-Third Edition (BSID-III) was used to assess the developmental level of patients between 12 and 42 months of age [26]. A composite score below 70 (2SD below the mean) in the cognitive indicates moderate of profound developmental delay; a score in the cognitive below 85 (1SD below the mean) indicates mild impairment; a score in the cognitive of 100 or more indicates normal. The Wechsler preschool and primary scale of intelligence-fourth edition (WPPSI-IV) was used to assess the development level of children more than 42 months of age (normal (FSIQ (full scale IQ score) ≥ 70); mild (FSIQ 50–69); moderate or profound (FSIQ < 50)).

Prognosis was graded into three categories according to different evaluation scores:

- 0: normal group (any evaluation score was normal)
- 1: mild impairment group (any evaluation score was mild)
- 2: encephalopathy group (any evaluation score was moderate or profound, Including patients with frequent convulsions and underdevelopment at the time of death)

### Statistics

Normally distributed data are presented as means± standard deviation (SD), skewed data are presented as medians (interquartile range).

Prognosis was regressed on predictors with ordinal logistic model. Considered variables include gender, gestational age, birth weight, age of the initial seizures, early interictal VEEG, variant location, delivery type. We modelled gestational age, birth weight, and age of the initial seizures as continuous variables, early interictal VEEG as ordinal variables which is analyzed as continuous data, and variant location, and delivery type as categorical variables. Predictors significant at α level 0.05 in univariate analyses were entered into a multivariate model and retained at the significance level of 0.05. Statistical analysis was performed by SPSS statistics version 21.0.

## Results

Fifty-seven infants (from Jan 2016 to Mar 2020) who had onset of seizures within the first month of life were enrolled. All of them carry *KCNQ2* gene pathogenic variants confirmed by molecular genetic testing in Children’s Hospital of Fudan University. Three infants were excluded due to unavailable VEEG data in the neonatal period, and two infants were excluded because of lost to follow-up.

Of the 52 infants included, 23 (44.2%) were females. The clinical features of the 52 patients range from BFNS1 (normal group) at mild end to EIEE7 (encephalopathy group) at severe end. The demographic data and clinical features and *KCNQ2* variation information are summarized in Table 1 and Table 2.

**Table 1:** Demographic data and clinical features of the patients

variable	Category	Long-term outcome (*n* = 52)		
		Normal group (*n* = 17)	Mild impairment group (*n* = 2)	encephalopathy group (*n* = 33)
		n (%)	n (%)	n (%)
Sex	Male	7 (41.18)	1 (50)	21 (63.34)
	Female	10 (58.82)	1 (50)	12 (36.36)
Birth weight(g)	Median (interquartile range IQR)	3350 (3045–3665)	/	3150 (2908–3600)
Gestational age (weeks)	Median (IQR)	39.43 (39.00–40.00)	/	39.43 (38.00–40.00)
Age of initial seizures (days)	Median (IQR)	3 (2.5–4.0)	/	2 (1.5–3.5)
Score of Early interictal VEEG	0	6 (35.29)	0	0
	1	5 (29.41)	1 (50)	0
	2	5 (29.41)	1 (50)	2 (6.06)
	3	1 (5.9)	0	10 (30.30)
	4	0	0	21 (63.64)
Variant location	Key spots	3 (17.65)	1 (50)	22 (66.67)
	Non-key spots	14 (82.35)	1 (50)	11 (33.33)
Delivery type	Vaginal delivery	9 (52.94)	1 (50)	16 (48.48)
	Cesarean section	8 (47.06)	1 (50)	17 (51.52)

/: not applicable because *n* = 2

**Table 2:** Clinical features of 52 patients with KCNQ2-related disorder

Patient	Gender	Age at onset (day)	Follow-up time (months)	Development	Variants	Location	Type of variants	GnomAD/1000gnome	Clinvar/HGMD	Reference	Familial targeted variantion study
1	M	2	26	C	NM_172107:exon15:c.1632 T > G(p.C544W)	C-terminal region	missense	0/0	/	/	De novo
2	M	2	28	C	NM_172107:exon8:c.1064A > T(p.D355V)	C-terminal region	missense	0/0	/	/	De novo
3	F	10	26	C	NM_172107:exon1:c.205del(p.K69Qfs*51)	N-terminal region	frameshift	0/0	Pathogenic/DM	PMID 14985406	Paternal
4	M	13	21	D	NM_172107:exon4:c.638G > A(p.R213Q)	voltage-sensor helix /S4	missense	0/0	Pathogenic/DM	PMID 22275249	/
5	F	4	25	B	NM_172107:exon14:c.1609A > T(p.K537X)	C-terminal region	stop_gained	0/0	Pathogenic/DM	PMID 24375629	De novo
6	M	4	16	A	NM_172107:exon4:c.553G > A(p.A185T)	S3	missense	0/0	/	/	De novo
7	M	13	30	C	NM_172107:exon15:c.1657C > T(p.R553W)	C-terminal region	missense	0/0	Pathogenic/DM	PMID 23621294	/
8	F	1	12	A	NM_172107:exon17:c.2127del(p.V710Cfs*155)	C-terminal region	frameshift	0/0	Pathogenic/DM	PMID 29655203	/
9	F	3	39	A	NM_172107:exon13:c.1342C > T(p.R448X)	C-terminal region	stop_gained	0/0	Pathogenic/DM	PMID 11690625	/
10	F	2	18	D	NM_172107:exon4:c.638G > A(p.R213Q)	S4	missense	0/0	Pathogenic/DM	PMID 22275249	De novo
11	F	4	24	A	NM_172107:exon14:c.1631 + 1G > A	C-terminal region	splice_region	0/0	Pathogenic/DM	PMID 25982755	/
12	F	3	40	A	NM_172107:exon14:c.1601del(p.P534Rfs*31)	C-terminal region	frameshift	0/0	Likely Benign/NA	/	De novo
13	F	3	39	B	NM_172107:exon8:c.1040A > G(p.Y347C)	C-terminal region	missense	0/0	/	/	De novo
14	F	3	50	A	NM_172107:exon13:c.1452G > A(p.W484X)	C-terminal region	stop_gained	0/0	/	/	De novo
15	F	4	30	A	NM_172107:exon8:c.1027G > T(p.A343S)	C-terminal region	missense	0/0	/	/	Maternal
16	F	1	18	A	NM_172107:exon2:c.365C > T(p.S122L)	the extracellular domain between the S1 segment and the S2 segment	missense	0/0	Pathogenic/DM	PMID 16916607	/
17	F	7	38	A	NM_172107:exon11:c.1230del(p.P411Rfs*29)	C-terminal region	frameshift	0/0	NA/DM	/	Paternal
18	M	9	12	A	NM_172107:exon4:c.668C > T(p.S223F)	Cytoplasmic between S4 segment and S5 segment	missense	0/0	/	/	De novo
19	F	3	13	A	NM_172107:exon4:c.620G > A(p.R207Q)	S4	missense	0/0	Pathogenic/DM	PMID 17872363	De novo
20	M	3	16	A	NM_172107:exon1:c.242 T > C(p.L81P)	N	missense	0/0	NA/DM	PMID 29215089	/
21	M	3	42	A	NM_172107:exon5:c.816 + 1G > A	Pore loop/H5	splice_region	0/0	Likely pathogenic/NA	/	De novo
22	M	2	14	D	NM_172107:exon5:c.794C > T(p.A265V)	H5	missense	0/0	Pathogenic/DM	PMID 22926866	/
23	F	1	15	D	NM_172107:exon5:c.796G > C(p.D266H)	H5	missense	0/0	/	/	De novo
24	M	2	5(deceased)	D	NM_172107:exon5:c.793G > A(p.A265T)	H5	missense	0/0	Pathogenic/DM	PMID 23692823	/
25	M	12	16	D	NM_172107:exon4:c.617 T > G(p.L206R)	S4	missense	0/0	/	/	De novo
26	M	4	49	B	NM_172107:exon4:c.587C > T(p.A196V)	S4	missense	0/0	Pathogenic/DM	PMID 17475800	De novo
27	M	2	36	C	NM_172107:exon4:c.637C > T(p.R213W)	S4	missense	0/0	Pathogenic/DM	PMID 18353052	De novo
28	M	2	7 (deceased)	D	NM_172107:exon4:c.629G > A(p.R210H)	S4	missense	0/0	Pathogenic/DM	PMID 24107868	/
29	F	4	28	D	NM_172107:exon4:c.632 T > G(p.M211R)	S4	missense	0/0	/	/	De novo
30	F	2	24	A	NM_172107:exon7:c.997C > T(p.R333W)	C-terminal region	missense	0/0	NA/DM	PMID 16039833	De novo
31	M	13	24	D	NM_172107:exon5:c.715G > C(p.G239R)	Pore domain/S5	missense	0/0	Pathogenic/DM	PMID 23692823	De novo
32	M	1	12	C	NM_172107:exon5:c.749 T > G(p.V250G)	S5	missense	0/0	Pathogenic/DM	PMID 11690625	De novo
33	M	3	14	C	NM_172107:exon5:c.794C > T(p.A265V)	H5	missense	0/0	Pathogenic/DM	PMID 22926866	De novo
34	M	24	12	D	NM_172107:exon4:c.568A > T(p.N190Y)	the extracellular domains between the S3 segment and the S4 segment	missense	0/0	/	/	De novo
35	F	1	52	D	NM_172107:exon5:c.781 T > A(p.F261I)	Pore loop	missense	0/0	/	/	De novo
36	F	3	14	D	NM_172107:exon15:c.1678C > T(p.R560W)	C-terminal region	missense	0/0	Pathogenic/DM	PMID 22275249	De novo
37	F	2	25	C	NM_172107:exon15:c.1678C > T(p.R560W)	C-terminal region	missense	0/0	Pathogenic/DM	PMID 22275249	/
38	F	3	22	D	NM_172107:exon15:c.1687G > A(p.D563N)	C-terminal region	missense	0/0	Pathogenic/DM	PMID 26007637	De novo
39	F	1	40	C	NM_172107:exon17:c.2331del(p.E778Rfs*152)	C-terminal region	frameshift	0/0	Likely benign-related/NA	/	De novo
40	M	2	50	D	NM_172107:exon8:c.1049A > T(p.N350I)	C-terminal region	missense	0/0	Likely pathogenic/NA	/	De novo
41	M	11	12	C	NM_172107:exon15:c.1687G > A(p.D563N)	C-terminal region	missense	0/0	Pathogenic/DM	PMID 26007637	De novo
42	M	3	12	A	NM_172107:exon3:c.484_485delAA(p.K162Vfs*10)	Cytoplasmic between S2 segment and S3 segment	frameshift	0/0	/	/	Paternal
43	M	2	12	C	NM_172107:exon6:c.821C > T(p.T274M)	H5	missense	0/0	Pathogenic /DM	PMID 22275249	/
44	M	1	16	A	NM_172107:exon4:c.650C > T(p.T217I)	S4	missense	0/0	Likely pathogenic/NA	/	De novo
45	M	2	12	C	NM_172107:exon5:c.807G > T(p.W269C)	H5	missense	0/0	NA/DM-related	PMID 14534157	De novo
46	F	1	54	C	NM_172107:exon5: c.794C > T(p.A265V)	H5	missense	0/0	Pathogenic/DM	PMID 22926866	De novo
47	M	7	37	C	NM_172107:exon5:c.715G > C(p.G239R)	S5	missense	0/0	Pathogenic/DM	PMID 23692823	De novo
48	F	2	46	C	NM_172107:exon4:c.602G > A(p.R201H)	S4	missense	0/0	Pathogenic/DM	PMID 23708187	De novo
49	M	2	63	C	NM_172107: exon4: c.637C > T(p.R213W)	S4	missense	0/0	Pathogenic/DM	PMID 18353052	De novo
50	M	3	12	C	NM_172107:exon15:c.1678C > T(p.R560W)	C-terminal region	missense	0/0	Pathogenic/DM	PMID 22275249	De novo
51	F	1	24	C	NM_172107:exon5:c.740C > T(p.S247L)	Pore domain/S5	missense	0/0	Pathogenic/Likely pathogenic​/DM-related	PMID 16916607	De novo
52	M	2	12	C	NM_172107:exon6:c.822_831delinsC(p.L275_T277del)	H5	Microsatellite	0/0	NA/DM-related	/	De novo

M = male; F = female; A = normal group; B = mild impairment group; C = encephalopathy group, including patients deceased; NA or /= no relevant information

Birth weight of the subjects was 3.31 ± 0.47 kg, with gestational age of 39.14 ± 1.41 weeks. The median date of initial seizures was 2.5 (IQR 2–4) days after birth of life. Half subjects were followed up after 24 (IQR 14–38) months of age (excluding two patients deceased). Forty-three infants between 12 and 42 months of age were assessed by BSID-III, 7 infants between 48 and 63 months of age were assessed by WPPSI-IV, 2 infants who didn’t undergo any detailed neurologic testing had frequent convulsions and underdevelopment at the time of death. Seventeen infants had normal development, 2 infants had mild impairment development, 33 infants had moderate/severe impairment development including 2 infants deceased. Univariable predictors are presented in the Table 3. Two variables including early interictal VEEG (β = 2.77, 1.20 to 4.34, P = 0.001) and variant location (β = 2.77, 0.03 to 5.5, P = 0.048) were statistically significant associated with prognosis. The result of multivariate analysis was shown in the Table 2. The results of multivariate analysis showed that early interictal VEEG (β = 2.77, 1.20 to 4.34, *P* = 0.001), and variant location (β = 2.77, 0.03 to 5.5, *P* = 0.048) were independent risk factors for poor long-term outcome.

**Table 3:** Univariable analysis and multivariable analysis

Univariable analysis			
Variables	β (95%CI)	SE	*P* value
Early interictal VEEG	2.72(1.28, 4.16)	0.74	0.00
Variant location	2.06(0.75, 3.38)	0.67	0.00
Gender	−0.88(−2.02, 0.26)	0.58	0.131
Birth weight(g)	−0.00(− 0.00, 0.00)	0.00	0.143
Gestational age	−0.14(− 0.56, 0.281)	0.21	0.52
Age of initial seizures	0.06(− 0.09, 0.21)	0.08	0.43
Delivery type	0.17(−0.95, 1.29)	0.57	0.77
Multivariable analysis			
Variables	β (95%CI)	SE	P value
Early interictal VEEG	2.77(1.20, 4.34)	0.80	0.001
Variant location	2.77(0.03, 5.5)	1.40	0.048

## Discussion

Early interictal VEEG is statistically significant in judging prognosis. The worse the early interictal VEEG, the worse the prognosis. VEEGs of the patients with *KCNQ2*-related epileptic encephalopathy showed a BS pattern, hypsarrhythmia, or multifocal epileptiform activity in our study, which is similar to reported studies [2, 4, 6–10, 12]. In other studies, VEEG of patients with BFNS1 appears normal or rarely showing a pattern of “theta pointu alternant” [1]. VEEG of patients with BFNS1 showed normal or mild multifocal sharp waves in our study. Multifocal epileptiform activity also can be observed in the patients with normal outcome or mild impairment development in our study. So, what is the difference of the multifocal epileptiform activity between encephalopathy infants and BFNS1 infants? In patients with moderate or severe impairment development, the sleep cycle of the VEEG often was indistinguishable. While in the patients with normal or mild impairment development, the VEEGs usually show existing of sleep cycle. Therefore, we speculate that the long-term outcome is positively correlated with the discharges of the early interictal VEEG. Interictal VEEG is useful for predicting long-term prognosis in our study. An article also revealed the ictal and interictal amplitude-integrated electroencephalogram (AEEG) and EEG play a certain role in the diagnosis of *KCNQ2*-related disorder early, and showed diversity characteristics of EEG at different variant locations [19]. Another article reported ictal amplitude-integrated electroencephalography pattern in newborns with neonatal epilepsy associated with KCNQ2 variants, the graphics are similar to Fig. 1.D in our article, the patients with this EEG pattern also has a moderate or severe impairment development [27].

Most patients with KCNQ2-related disorder were self-limited epilepsy, but most of them had a severe impairment development. Some articles [18, 19, 27] suggested that using sodium channel blockers as the first-line treatment can improve the prognosis of some patients. So we speculate that effective control of convulsions during the critical period of brain development is an effective measure for a good prognosis. It is possible that VEEG monitoring after using anti-epileptic drugs is more sensitive to predict the prognosis. In our study, phenobarbital was the first-line treatment for seizures in the neonates of our cohort. If the convulsions were not controlled, multiple anti-epileptic drugs were treated subsequently. For example, patient 34 had a refractory epileptic encephalopathy, the score of the first-time VEEG data was 3 without using any AEDs, while the score of the VEEG data 7 days later was 4 after using phenobarbital; patient 5 had mild intellectual development, the score of the first time VEEG data was 3 without using any AEDs, while the score of the VEEG data 5 days later was 2 after using phenobarbital. Patients whose VEEG show a BS pattern, hypsarrhythmia, or multifocal epileptiform activity should have a consideration for the early use of sodium channel blockers before the genetic confirmation.

Patients of our study also experienced seizure frequency declined over the years as reported in the literatures [28], and recurrent seizures often occurring after seizure-free for at least 2 years are reported in a recent study [28]. This phenomenon were not observed in our group for the median age at follow-up was 24 months of age, so some infants still had convulsions, meanwhile most infants had not yet reached the age of recurrence seizures. The reasons for the seizure frequency declining over the years and the recurrence of convulsions are not yet clear. We know that KCNQ2 gene is expressed predominantly during embryonic and adult stage from Uniprot database. This phenomenon of the seizure frequency declining over the years and the recurrence of convulsions may be related to the time of gene expression.

Variant location is statistically significant in predicting a negative prognosis, patients with pathogenic variants in the pore-lining domain or S4 segment has a worse outcomes. A recent article did not identify a relationship between variant position and cognitive outcome in patients harboring missense variants [29], the researchers in this study divided location into two group: (1) transmembrane variants; (2) variants located in the C terminus and the different loops. Location grouping is different from our group. We divided location into two groups: (1) S4 segment and the pore-lining domain (from S5 to S6, including S5,S5-H5 linker, H5,H5-S6 linker, S6); (2) not meet the previous one. It has been found that pathogenic variants in BFNS1 occur throughout the gene, including exon and whole-gene deletions; pathogenic variants in EIEE7 cluster in four functionally important protein domains: voltage sensor, the pore, C-terminus proximal region (important for modulation by second messengers), and calmodulin-binding B helix region [11, 30]. One recent study showed “severe or epileptic encephalopathy (EE)” missense variants cluster at S4, the pore loop that contains the selectivity filter, S6, helix B, and the helix B-C linker [31]; another reported that the EE missense variants cluster at the pore domain, S6, and pre-helix A [32]. The above literatures also show that the variants of encephalopathy is prone to occur in the pore-lining domain or S4 segment.

In this study, long-term outcome was usually worse when the variant location was in voltage sensor and the pore domain even early VEEG score was same. For example, patient 26 with a variant in S4 segment with early VEEG score 1, had a mild impairment development, while other patients with early VEEG score 1 usually had a normal development; patient 27 with a variant in S4 segment with early VEEG score 2 had a moderate or severe impairment development, while other patients with early VEEG score 2 usually had a normal or mild impairment development. Correspondingly, the long-term outcome was relatively mild when variant location was not in voltage sensor and the pore domain. For example, patient 42 with a variant in S2-S3 linker with early VEEG score 3 had a normal development, while other patients with early VEEG score 3 usually had a moderate or severe impairment development. So the relationship between variant position and outcome needs more data and further research.

According to the prognosis, we found that there were few patients with KCNQ2-related disorder whose outcome was between benign and encephalopathy. The intellectual development of these patients was slightly impaired. The early VEEG of these patients showed multifocal epileptiform activity with normal wake cycles. The number of abnormal discharges was more than that of the patients with normal outcomes, but less than encephalopathy ones. This intermediate type between benign and encephalopathy has also been reported in other researches [11, 12, 16]. Some studies defined this group as benign, some put them into encephalopathy group. In our study, we believe it should be defined as a new independent group.

It is well known that same variant in KCNQ2 can cause different phenotypes varying from mild forms to severe forms. Similarly, in our study, patient with similar VEEG have different prognosis, but patients with variants located in S4 segment or the pore-lining domain usually has a worse outcome. Therefore, it is not enough to evaluate a patient’s prognosis by relying solely on the location or VEEG. Both variant location and VEEG should be combined to predict the prognosis. *KCNQ2*-related disorder is a rare disease, a cohort of 52 cases is a large sample size for a single-center study. Besides, it still needs to be supported by more samples from multi-center or long-term follow-up data.

## Conclusion

Two combined clinical characteristics, VEEG and variant location, provide clinicians a method for assessing prognosis of *KCNQ2*-related disorder. A neonate with a *KCNQ2* variant location in voltage sensor or the pore-lining domain, worse VEEG background, usually had a poor prognosis.

## Data Availability

The datasets used and/or analysed during the current study available from the corresponding author on reasonable request.

## Abbreviations

BFNS1: benign familial neonatal seizures 1
EIEE7: early infantile epileptic encephalopathy 7
VEEG: video-electro-encephalography
AEDs: anti-epileptic drugs
FSIQ: full scale IQ
SD: standard deviation
IQR: interquartile range
EE: epileptic encephalopathy
